# Does handwriting the name of a potential trial participant on an invitation letter improve recruitment rates? A randomised controlled study within a trial

**DOI:** 10.12688/f1000research.18939.1

**Published:** 2019-05-14

**Authors:** Jennifer McCaffery, Alex Mitchell, Caroline Fairhurst, Sarah Cockayne, Sara Rodgers, Clare Relton, David J. Torgerson, OTIS Study Team

**Affiliations:** 1 Department of Health Sciences, University of York, York, North Yorkshire, YO10 5DD, UK; 2 SCHARR, University of Sheffield, Sheffield, South Yorkshire, S1 4DA, UK

**Keywords:** Recruitment, Randomised Controlled Trial, Embedded trial, Personalisation, Handwritten

## Abstract

**Background: **Randomised controlled trials (RCTs) often fail to recruit to target, resulting in a lack of generalisability of findings. A wide range of strategies for potentially increasing recruitment have been identified; however, their effectiveness has not been established. The aim of this study within a trial (SWAT) was to evaluate the effectiveness of handwritten personalisation of an invitation letter as part of a trial recruitment pack on recruitment to a host RCT.

**Methods:** A pragmatic, two-armed RCT was conducted, embedded within an existing falls prevention trial (OTIS) in men and women aged 65 years and over living in the community. Participants were randomised 1:1 to receive an OTIS recruitment pack containing an invitation letter on which their name was handwritten (intervention group), or one on which it was printed (control group). The primary outcome was randomisation into the host trial.  Secondary outcomes related to trial eligibility and retention.  Analyses were via logistic regression and Cox Proportional Hazards regression.

**Results: **Of the 317 SWAT participants, 12 (3.8%) were randomised into the OTIS trial: 3 (handwritten: 3/159 [1.9%]; printed: 9/158 [5.7%]; difference -3.8%, 95% CI -8.0% to 0.4%). There was weak evidence, against the intervention, of a difference in the likelihood of participants being randomised into the host trial between the two groups (OR 0.32, 95% CI 0.08 to 1.20, p=0.09). There were no statistically significant differences between the intervention and control groups on any of the secondary outcomes.

**Conclusions:** There was no evidence that personalisation of invitation letters improved recruitment to the OTIS trial. However, due to the small sample size, the results should be interpreted with caution. These findings need to be replicated across larger studies and wider populations.

**Registration:** ISRCTN22202133.

## Introduction

Randomised controlled trials (RCTs) are regarded as the gold standard design to evaluate the effectiveness of interventions in health research ^1– 3^. However, a recent review of RCTs funded by the National Institute of Health Research (NIHR) in the UK found that only 56% of RCTs reviewed achieved their planned sample size ^4^.

Trialists are well aware of recruitment challenges and have adopted a wide range of strategies to achieve and retain their sample size, including gifts, reminders and enhanced cover letters ^5, 6^. A recent Cochrane review has evaluated strategies used to improve recruitment to RCTs ^7^. This review identified 68 trials involving more than 74,000 participants. The key conclusions from this review were that there was high-certainty evidence for three methods. The first is that informing participants what they will receive in the trial improves recruitment. The second is that phoning people who do not respond to postal invitations can be effective. Finally, using a tailored, user-tested information sheet makes little or no difference to recruitment. The review found that of the 72 strategies evaluated, only seven involved more than one study; therefore, additional studies are needed to evaluate the effectiveness of strategies to improve recruitment.

One potential strategy that has been found to be effective at improving the responses to postal questionnaires ^8^ is making trial documentation more personal. In a review by Edwards *et al*. ^8^, data from 58 trials demonstrated that the odds of returning a questionnaire was increased by more than one tenth (OR 1.14, 95% CI 1.07 to 1.22) when questionnaire material was made more personal. However, there was a wide range of ‘personalisation’ investigated within these trials (e.g. hand-addressing envelopes, signing letters personally etc.). Another systematic review looked specifically at the effect of personally addressed and/or hand-signed letters on questionnaire response. This review found that there was a positive effect on response rates when letters were personally addressed, which increased when letters were also hand-signed ^9^.

Our aim was to undertake a study within a trial (SWAT) to determine whether the number of participants recruited to a trial can be improved by writing the potential participant’s name by hand, versus printing the name, on the invitation letter. By embedding a study within a RCT currently being coordinated by the York Trials Unit (YTU; University of York), the NIHR-funded OTIS study ^10^, the effects of the intervention could be tested within a pragmatic context without the additional costs of participant recruitment.

## Methods

### Ethics approval

This trial was embedded within the NIHR Health Technology Assessment (HTA) funded Occupational Therapist Intervention Study (OTIS) ^10^ (Programme grant number 14/49/149). The OTIS study is an RCT that aims to evaluate the clinical and cost effectiveness of an occupational therapist (OT) intervention to reduce falls in high risk older people. Ethical approval for the OTIS trial and this embedded study was given by the West of Scotland Research Ethics Service (WoSRES); Health Research Authority approval and the Department of Health Sciences Research Governance Committee at the University of York. This study within a trial was registered with the ISRCTN registry as part of the host trial registration (ISRCTN22202133; date registered: 20 ^th^ June 2016)

### Participant recruitment

**Figure 1.**
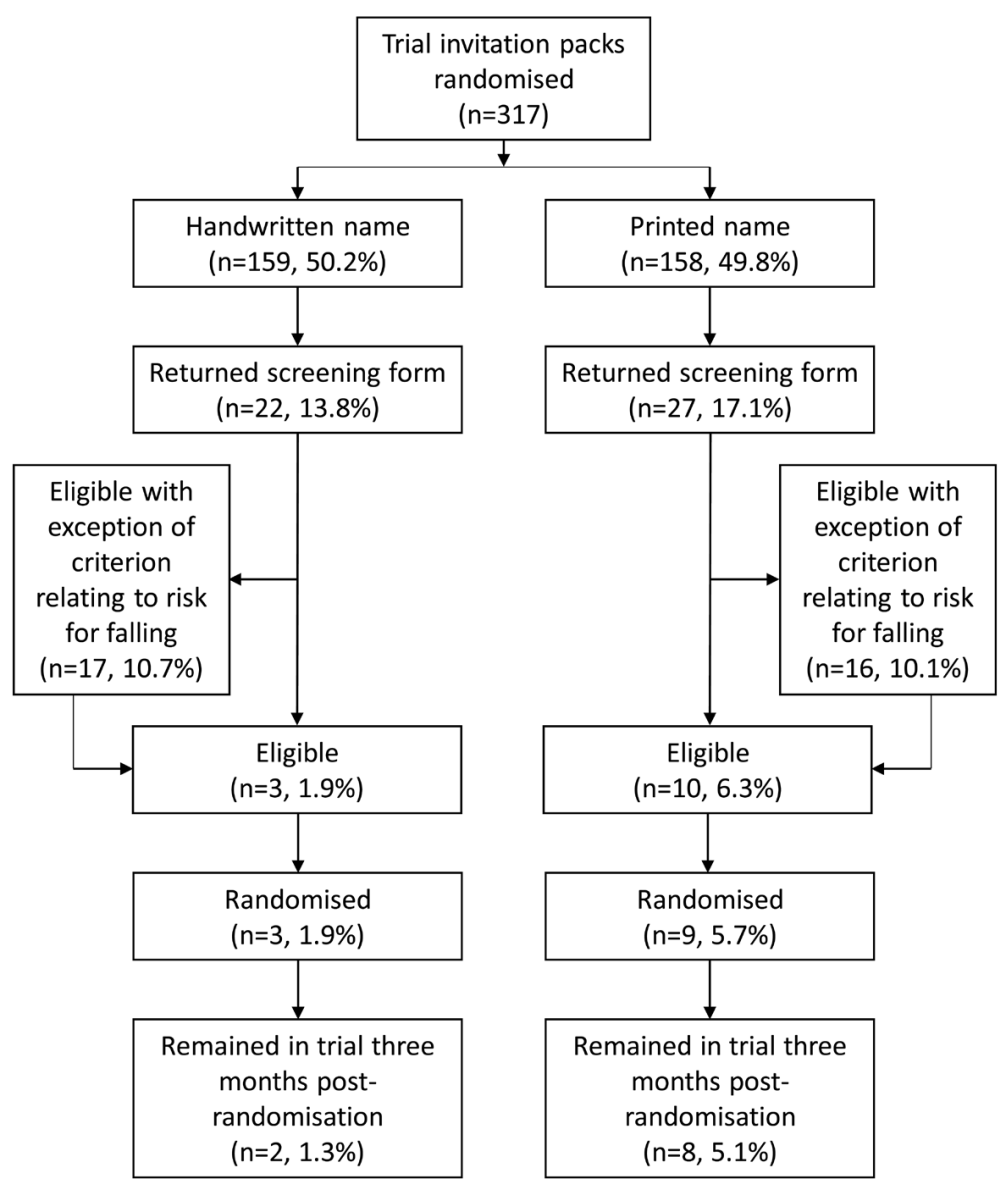
Participant flow diagram.

The OTIS trial is a modified cohort ^11^, pragmatic, multicentre, two-armed RCT. Detailed methods of the main trial have been published elsewhere ^10^. Participants for OTIS were recruited by mail out of recruitment packs to: i) participants in the Yorkshire Health Study (YHS) ^12^; ii) cohorts of previous trials held by the YTU ^13– 15^; iii) potentially eligible patients identified in GP practice databases; and iv) via opportunistic screening. This embedded trial involved potential participants who were approached in the first mail out from the YHS (see Figure 1 for a participant flow diagram). Recruitment to the embedded trial began on the 20 ^th^ of April 2017 and follow-up ended on the 22 ^nd^ August 2018.

After receiving a recruitment pack (consisting of an invitation letter, participant information sheet, consent form, contact form and screening questionnaire) participants were asked to return their completed screening questionnaire and consent form to researchers based at the YTU if they wished to take part in the study. Participants were eligible to take part in the OTIS trial if they were aged 65 years or over, lived in the community, had fallen in the past 12 months or had a fear of falling, and were willing to receive a home visit from an OT. Participants were ineligible if they were unable to walk 10 feet (even with a walking aid), had dementia, lived in a residential or nursing home, had poor levels of English (with no access to assistance), had received an OT assessment for falls prevention in the last 12 months or were on a waiting list for an assessment, or had not returned a completed falls calendar. Participants who were eligible except for the fact that they had not fallen in the last 12 months and did not report a fear of falling were rescreened at intervals until they asked not to continue participation or they became eligible. Once eligible, participants were sent a baseline questionnaire and pack of falls calendars. Participants who completed their baseline questionnaire and falls calendars then became eligible for randomisation into the OTIS trial. For the main OTIS trial, participants were randomised to an environmental assessment by an OT or to the control group.

## Randomisation and blinding

This trial was embedded in the OTIS trial, and included potential OTIS participants sent a recruitment pack as part of the first mail out to consenting members of the YHS. Recruitment packs were assigned a unique identification number. Block randomisation was used to allocate the recruitment packs in a 1:1 ratio to either the control group or the intervention group, using one large block the size of the mail out (n=317). Generation of the allocation sequence was undertaken by the OTIS trial statistician, who was not involved with the production of the recruitment packs, using Stata version 13.

### Control group

The control group received the standard recruitment pack with an invitation letter with their name printed in the salutation. The letter was printed on one page of A4 and is shown in *Extended data*, Supplementary File 1 ^16^.

### Intervention group

The intervention group received the standard recruitment pack with an invitation letter with their name handwritten in the salutation. The names were written by a researcher in in the form Mr/Ms Firstname Surname. The letter was printed on one page of A4 and is shown in *Extended data*, Supplementary File 2 ^16^.

### Primary outcome

The primary outcome was the proportion of participants included in the embedded trial, who went on to be randomised to the host OTIS trial.

### Secondary outcomes

The secondary outcomes were:

- proportion of participants who returned a screening form
- time to return screening form
- proportion of participants who fulfilled the eligibility criteria on their initial screening form apart from the criterion relating to falls within past 12 months or fear of falling
- proportion of participant who were eligible on their initial screening form
- proportion of participants who remained in the trial at three months post randomisation (defined as returning at least the first three months’ worth of falls calendars from the date of randomisation).

### Sample size

We randomised 317 participants who were due to be mailed out a recruitment pack about the OTIS trial by the YHS. This sample size is sufficient to detect a 10% absolute difference in the percentage of participants who go on to be randomised (from 10 to 20%) between the two groups at 80% power and a two-sided alpha level of 0.1.

### Statistical analysis

Data were analysed on the basis of intention-to-treat and all hypothesis tests were two-sided at the 10% significance level. Categorical data were compared using logistic regression models and time to response data using a Cox proportional hazards model. An additional logistic regression model used to analyse whether participants remained in the trial 3 months post-randomisation was adjusted for the OTIS main trial group allocation (usual care or intervention). The odds ratio (OR) or hazard ratio (HR) from each model associated with the embedded trial allocation is presented along with the corresponding 95% confidence interval (CI) and p-value. All analyses were conducted using Stata version 15.

A completed CONSORT checklist is available at Open Science framework ^16^.

## Results

### Primary outcome

***Randomised to OTIS main trial.*** Of the 317 embedded trial participants, 12 (3.8%) were randomised into the OTIS trial (handwritten: 3/159 [1.9%]; printed: 9/158 [5.7%]; difference -3.8%, 95% CI -8.0% to 0.4%). There was weak evidence of a difference in the likelihood of embedded trial participants being randomised into OTIS between the two groups (OR 0.32, 95% CI 0.08 to 1.20, p=0.09), in favour of the control group.

### Secondary outcomes

***Returned screening form.*** In total, 49 (15.5%) of the 317 embedded trial participants returned a screening form (handwritten: 22/159 [13.8%]; printed: 27/158 [17.1%]; difference -3.3%, 95% CI -11.2% to 4.7%). There was no evidence of a difference in the likelihood of embedded trial participants returning a screening form between the two groups (OR 0.78, 95% CI 0.42 to 1.44, p=0.42).

***Time to return of screening form.*** For the screening forms returned, the median time to return was 26 days (interquartile range [IQR] 20 to 60) in the handwritten arm and 26 days (IQR 20 to 204 days) in the printed arm. There was no evidence of a difference in the time to response between the two arms (HR 0.81, 95% CI 0.46 to 1.41, p=0.45).

***Participants eligible apart from criterion relating to falls.*** Of the 317 embedded trial participants, 33 (10.4%) were initially ‘almost’ eligible for the OTIS trial (handwritten: 17/159 [10.7%]; printed: 16/158 [10.1%]; difference 0.6%, 95% CI -6.2% to 7.3%). There was no evidence of a difference in the likelihood of participants being eligible on initial screen except for the risk factors for falling between the two groups (OR 1.06, 95% CI 0.52 to 2.19, p=0.87).

***Participants eligible for trial.*** In total, 13 (4.1%) of the 317 embedded trial participants were eligible for the OTIS trial on their initial screening form (handwritten: 3/159 [1.9%]; printed: 10/158 [6.3%]; difference -4.4%, 95% CI -8.8% to -0.1%). There was weak evidence of a difference in the likelihood of participants being fully eligible on initial screen between the two groups (OR 0.28, 95% CI 0.08 to 1.05, p=0.06), in favour of the control group.

***Participants who remained in the trial at 3 months post randomisation (return first three falls calendars).*** Of the 317 embedded trial participants, 10 (3.2%) remained in the OTIS trial 3 months post-randomisation (handwritten: 2/159 [1.3%]; printed: 8/158 [5.1%]; difference -3.8%, 95% CI -7.6% to 0%). There was some evidence of a difference in the proportion of embedded trial participants remaining in the main OTIS trial between the two groups in favour of the control group (unadjusted OR 0.24, 95% CI 0.05 to 1.14, p=0.07). When the logistic regression was adjusted for main trial allocation (which reduced the included sample size to 12) the size of the effect was similar but the confidence interval was much wider and the p-value larger (handwritten: 2/3 [66.7%]; printed: 8/9 [88.9%]; adjusted OR 0.23, 95% CI 0.01 to 5.93, p=0.37).

## Discussion

We found that recipients of a personalised invitation letter, on which their name had been handwritten, were three times less likely to be randomised into the host OTIS trial, and this difference was statistically significant at the 10% level.

Finding that personalisation did not increase recruitment to the OTIS trial provides a significant contribution to the limited literature on improving randomisation and recruitment to RCTs. The outcomes of the trial do not support the review by Edwards *et al*. ^8^, which found that personalisation increased the odds of participants’ returning a questionnaire. However, not all of the studies included in the review observed an increase in response rate using personalisation. Moss and Worthern ^17^ found that writing the potential participant’s name by hand significantly reduced response rates when compared to typing the name. The review by Edwards *et al*. ^8^ included a wide range of studies within a wide range of contexts (e.g. teachers, students and individuals selected at random from the telephone directory). Moss and Worthern ^17^ invited psychologists to provide their views on standardised assessments. They suggest that the reduction in response rates may have been due to handwriting being perceived as more personal but less professional. This supports Linsky’s theory that there are complex interactions between methods used to increase recruitment and the context in which they are used ^18^. Our findings may also provide additional evidence for Linsky’s ^18^ recommendation that in contexts requiring higher levels of confidentiality and professionalism, such as health research, personalisation may be contra-indicated.

There are some limitations to the study. Only a small number of potential participants were recruited and randomised to the OTIS study, indeed far fewer than anticipated, and so the embedded trial is severely underpowered. This reduces the reliability of our findings. This highlights the value of repeating this investigation with larger sample sizes and reviewing findings across similar studies. Potential participants were limited to individuals over the age of 65 years living in the community, as such the results are only applicable to this population. Further studies should substantiate the study results in other populations.

## Conclusion

Our findings that personalisation does not improve recruitment lend weight to the argument that methods to improve recruitment may well be context specific. Given the small sample size the results should be interpreted with caution and highlight the need to replicate and extend this work across larger studies and wider populations.

## Data availability

### Underlying data

Open Science Framework: OTIS Invitation Letter SWAT. https://doi.org/10.17605/OSF.IO/KGH4S ^16^.

This project contains the underlying data in CSV and SAV format, with a variable key in CSV format.

### Extended data

Open Science Framework: OTIS Invitation Letter SWAT. https://doi.org/10.17605/OSF.IO/KGH4S ^16^.

This project contains the following extended data:

- Supplementary File 1. Invitation letter for the Control Group.
- Supplementary File 2. Invitation letter for the Intervention Group.

## Reporting guidelines

Open Science Framework: CONSORT checklist for study “Does handwriting the name of a potential trial participant on an invitation letter improve recruitment rates? A randomised controlled study within a trial”. https://doi.org/10.17605/OSF.IO/KGH4S ^16^.

Data are available under the terms of the Creative Commons Zero "No rights reserved" data waiver (CC0 1.0 Public domain dedication).
