# Supplementary material for: Does handwriting the name of a potential trial participant on an invitation letter improve recruitment rates? A randomised controlled study within a trial
Source: F1000Res. 2019 May 14;8:659. [Version 1] doi: 10.12688/f1000research.18939.1 (PMC6733374; doi:10.12688/f1000research.18939.1)
Supplement: Peer review history

# Peer review: Reviewer response for version 1

**DOI:** 10.5256/f1000research.20762.r52960

**Published:** 2019-08-29

**Authors:** Miles D. Witham

**Affiliations:** 1 AGE Research Group, NIHR Newcastle Biomedical Research Centre, Newcastle upon Tyne Hospitals Trust, Newcastle University, Newcastle upon Tyne, UK

This SWAT (Study within a Trial) is a useful addition to the literature on trials recruitment methodology, and is no less valuable for attempting to replicate a previously tested intervention.

 The methods are appropriately described and there are links to the intervention and comparator letters as well as to the underlying data.

 The numbers are small, but the results are reported appropriately and the small numbers are acknowledged in the discussion. Limitations are appropriately discussed and conclusions are appropriately cautious.

 Points for consideration:

- I would like to know who actually wrote the participant name on the letters and produced the mailout packs; in particular, it needs to be clear if they had any role in the SWAT or the main trial beyond generating the mailout packs? This would reassure readers that the analysis was masked and that the allocation of packs did not influence any other study processes. A statement about who was masked (analysis team, main study team) and who wasn’t masked would also be helpful.
- The choice of a 10% significance level is non-standard (but is not inappropriate given the small anticipated sample size) – please could the authors explain and justify this choice to readers though?
- Ref 16 in the reference list is a reference to this paper, but without the hyperlink to the supplementary data to which the in-text reference seems to apply. Could this be fixed please?

I confirm that I have read this submission and believe that I have an appropriate level of expertise to confirm that it is of an acceptable scientific standard.

# Peer review: Reviewer response for version 1

**DOI:** 10.5256/f1000research.20762.r50004

**Published:** 2019-06-18

**Authors:** Martin C Gulliford

**Affiliations:** 1 School of Population Health and Environmental Sciences, King's College London, London, UK

This is a useful trial within a trial to explore different invitation methods. The report clearly describes what was done and presents clear conclusions.

 Comments:

1. The intervention appears to be under-theorised as a behaviour change intervention. It might be useful to cross reference the behavioural insights approach (e.g. publications by Anna Sallis) as well as using the TIDIER checklist.
2. In the analysis, the paper presents both a difference in proportions and an odds ratio, though the P value is not given for the former. The odds ratio appears to be considered the primary analysis, though the difference in proportions is more relevant. It is not clear that both analyses are needed.
3. In the Discussion, where it reads 'and this difference was statistically significant at the 10% level.' The ASA guidelines suggest that bright lines such as 0.1 or 0.05 should not be used. This P value does not appear to provide evidence of any real effect. https://www.amstat.org/asa/files/pdfs/P-ValueStatement.pdf 
4. May want to reference the Cowie study. ^1^

I confirm that I have read this submission and believe that I have an appropriate level of expertise to confirm that it is of an acceptable scientific standard.

## References

[1] : Handwritten 'post-it' notes, questionnaire formats and response to a postal questionnaire survey. *International Journal of Epidemiology*.2011;40(1) : 10.1093/ije/dyq043 254-255 10.1093/ije/dyq043 20308038
